# Rare Genetic Variants Underlying Primary Immunodeficiency: Clinical, Pulmonary, and Genetic Insights from Two Pediatric Cases

**DOI:** 10.3390/genes16111247

**Published:** 2025-10-22

**Authors:** Nurgul Sikhayeva, Svetlana Volodchenko, Elena Kovzel, Aiganym Toleuzhanova, Aliya Romanova, Gulnar Tortayeva, Yelena Sagandykova, Marina Morenko, Aidos Bolatov, Ilyas Akhmetollayev, Anar Shakirova, Mariya Tagaeva

**Affiliations:** 1 National Center for Biotechnology, Korgalzhyn Highway 13/5, Astana 010000, Kazakhstan; romanovaaliya@gmail.com (A.R.); iliyas@mail.ru (I.A.); 2 National Holding «QazBioPharm», Korgalzhyn Highway 13/5, Astana 010000, Kazakhstan; 3 “University Medical Center” Corporate Fund, St. Kerey, Zhanibek Khandar Khanov 5/1, Astana 010000, Kazakhstan; svetlanasv888@mail.ru (S.V.); elena.kovzel@umc.org.kz (E.K.); aiga1999nym@gmail.com (A.T.); gulnart@bk.ru (G.T.); l.e.n.a.78@mail.ru (Y.S.); maya.10.90@mail.ru (M.T.); 4 Department of Pediatrics with Courses in Allergology, Immunology, Hematology, and Endocrinology, School of Medicine, “Medical University of Astana” NAO, Beibitshilik Street 49/A, Astana 010000, Kazakhstan; morenko_m.a@mail.ru; 5 Medical School, Shenzhen University, 3688 Nanhai Road, Shenzhen 518060, China; bolatovaidos@gmail.com; 6 Division of Strategic Development and Science, “Human Research & Development” LLP, Qabanbay Batyr Ave. 11/2, Astana 010000, Kazakhstan

**Keywords:** primary immunodeficiency, fbln5, Lax skin syndrome, ataxia-telangiectasia, pulmonary fibrosis, granulomatous skin lesions, whole-exome sequencing, pediatric rare diseases

## Abstract

Background/Objectives: Inborn errors of immunity (IEIs), formerly known as primary immunodeficiency disorders, are a heterogeneous group of genetic diseases characterized by recurrent infections and multisystem involvement. Although more than 500 distinct entities have been identified, reports from Central Asia remain scarce. This study describes two rare pediatric IEI cases from Kazakhstan, highlighting the importance of genomic diagnostics in underrepresented regions. Methods: Two unrelated male patients with early-onset recurrent infections and systemic complications were evaluated at the University Medical Center, Astana. Clinical and laboratory assessments included immunophenotyping, imaging, and histopathology. Whole-genome sequencing (WGS) was performed, followed by Sanger confirmation and segregation analysis when feasible. Variants were classified according to ACMG/AMP guidelines. Results: The first case involved a child with recurrent bronchopulmonary disease, pulmonary fibrosis, and connective tissue abnormalities, found to carry a novel homozygous *FBLN5:*c.53del frameshift variant consistent with autosomal recessive cutis laxa type 1A. The second case concerned an adolescent with progressive neurodegeneration, granulomatous skin lesions, and chronic pancreatitis, who was identified with a heterozygous pathogenic *ATM*:c.4828dup variant, confirming ataxia–telangiectasia. Both patients required lifelong subcutaneous immunoglobulin therapy. Consanguinity contributed to the genetic risk in the first case, while the second case demonstrated diagnostic delays that emphasized the value of genetic testing. Conclusions: These cases underscore the clinical heterogeneity of IEIs and illustrate the essential role of genomic diagnostics in elucidating atypical presentations. Documenting rare variants and unconventional phenotypes enhances global knowledge, elevates awareness in resource-limited regions, and emphasizes the necessity for early, multidisciplinary care and the enhancement of national registries for rare immunogenetic disorders.

## 1. Introduction

Inborn errors of immunity (IEIs), formerly termed primary immunodeficiency disorders, represent a heterogeneous group of genetically determined diseases caused by defects in distinct components of the immune system [1]. Clinically, they present with recurrent or severe infections, autoimmune manifestations, and increased cancer risk [2]. According to the International Union of Immunological Societies (IUIS), more than 500 IEIs have been described, reflecting the rapid expansion of knowledge on their genetic basis [3]. These conditions affect individuals across all ages, sexes, and ethnicities and may occur either as isolated immunodeficiencies or within syndromic phenotypes [4,5].

The clinical and genetic heterogeneity of IEIs poses major diagnostic challenges [6]. Modes of inheritance include autosomal recessive, autosomal dominant, and X-linked patterns, while disease severity can vary even within families [7]. Phenotypic variability often arises from modifier genes, epigenetic regulation, or environmental factors. Traditionally, diagnosis relied on clinical evaluation, immunological testing, and the exclusion of secondary causes [8]. However, these methods frequently failed to detect atypical or overlapping phenotypes. With the advent of whole-exome sequencing (WES), whole-genome sequencing (WGS), and targeted next-generation sequencing (NGS) panels, genetic confirmation has become more accessible and reliable [9]. Genetic diagnostics now enable precise classification, early interventions, personalized therapy, and genetic counseling, making them an essential component of IEI evaluation.

Specific syndromic IEIs highlight the complexity of overlapping clinical manifestations. Autosomal recessive cutis laxa type 1A results from mutations in *FBLN5*, encoding fibulin-5, a protein critical for elastic fiber assembly [10]. Affected patients often exhibit loose skin, cardiovascular malformations, pulmonary complications, and variable systemic involvement. Similarly, ataxia-telangiectasia, caused by mutations in the *ATM* gene, is characterized by progressive cerebellar ataxia, telangiectasia, immunodeficiency, and cancer susceptibility [11]. Both conditions exemplify the multisystem complexity and phenotypic heterogeneity of IEIs, in which respiratory, connective tissue, and immunological abnormalities may obscure diagnosis [12].

In Central Asia, rare immunogenetic disorders remain underreported. Limited access to genomic testing, delayed specialist referral, and restricted treatment availability complicate diagnostics. In addition, relatively high consanguinity rates in some populations increase the likelihood of autosomal recessive disorders, broadening the IEI spectrum encountered in clinical practice [1]. Case-based research from underrepresented regions thus provides valuable insights, enhances recognition among physicians, and contributes to building epidemiological knowledge. Documenting such cases also guides diagnostic and therapeutic strategies in resource-constrained settings [13].

The present report describes two rare and complex IEI cases in male patients from Kazakhstan. The first involves a child with autosomal recessive cutis laxa type 1A due to a novel pathogenic *FBLN5* variant, presenting with pulmonary fibrosis, immunodeficiency, and connective tissue manifestations. The second concerns an adolescent with a heterozygous pathogenic *ATM* variant, manifesting as ataxia-telangiectasia with granulomatous skin lesions, bronchiectasis, and progressive neurological decline. These cases underscore the diagnostic value of genomic sequencing with Sanger confirmation, while also highlighting the challenges of managing multisystem IEIs in underrepresented regions.

## 2. Case Presentations

### 2.1. Case Description 1

A 12-year-old male presented with a lifelong history of recurrent respiratory infections and progressive pulmonary complications. He was the first surviving child of his mother’s second pregnancy; his older sibling died in early infancy due to pneumonia. The patient was born prematurely at 36–37 weeks of gestation and had no perinatal complications. Recurrent upper and lower respiratory tract infections began in infancy and were associated with bronchopulmonary obstruction requiring repeated hospitalizations throughout early childhood.

Over time, the frequency and severity of infections increased, and by school age, the patient developed persistent respiratory insufficiency. Multiple comorbid clinical features gradually emerged, including cutis laxa with lax skin folds, congenital heart disease with pulmonary artery stenosis, primary hypothyroidism, right thyroid lobe cyst, inguinoscrotal hernia, and bronchial asthma. Supportive therapy included bronchodilators, corticosteroids, and management of respiratory exacerbations.

Quantitative serum immunoglobulin measurements (IgG, IgA, IgM) were initially unavailable because such tests were not routinely performed at the regional hospital where the patient first received medical care. Subsequent evaluation at a tertiary center confirmed hypogammaglobulinemia, prompting initiation of long-term subcutaneous immunoglobulin replacement therapy to reduce infection frequency and prevent further pulmonary deterioration.

### 2.2. Case Description 2

A 16-year-old male presented with a history of recurrent respiratory infections, progressive neurological impairment, and chronic cutaneous lesions. He was born prematurely at 36–37 weeks with a birth weight of 2900 g. Early development was complicated by ataxic gait and motor coordination deficits. By school age, progressive neurological decline and recurrent infections became evident.

He completed routine immunizations until five years of age, when vaccinations were suspended due to frequent infections. By 2016, a diagnosis of ataxia–telangiectasia was established. Over the following years, he developed granulomatous skin lesions, recurrent pneumonias, bronchiectasis, and signs of chronic pulmonary disease. Cardiac evaluation later revealed mild valvular insufficiency and borderline pulmonary hypertension.

At the time when sarcoidosis was considered in the differential diagnosis, serum ACE activity, lysozyme, and sIL-2R were not assessed. Chest imaging performed at that time demonstrated bronchiectasis and interstitial changes without mediastinal or hilar lymphadenopathy. Abdominal ultrasound showed no hepatosplenomegaly and no abdominal lymph node enlargement. Biomarker testing will be performed if subsequent clinical or imaging findings raise suspicion for systemic sarcoidosis.

Genetic testing in 2024 identified a heterozygous pathogenic ATM frameshift variant (c.4828dup, p.Arg1610Lysfs*3), confirming the diagnosis of ataxia–telangiectasia.

Management consisted of comprehensive supportive therapy, including respiratory and cardiovascular care, dermatological monitoring, and lifelong subcutaneous immunoglobulin replacement therapy.

## 3. Materials and Methods

### 3.1. Subjects

This investigation included two unrelated male pediatric subjects who presented with recurrent severe infections, congenital malformations, and suspected primary immunodeficiency syndromes. Participants were recruited at the University Medical Center (Astana, Kazakhstan) after obtaining written informed consent from parents/guardians (assent was obtained from minors when applicable). Clinical data were collected using standardized forms and chart review, including perinatal history, immunization records, growth and developmental milestones, prior surgical interventions, family history (including parental consanguinity and immune or respiratory disorders), occurrence of infections, hospitalizations, and chronic comorbidities. Physical examination focused on dermatologic, pulmonary, cardiovascular, and neurologic systems.

Inclusion criteria comprised: (i) early-onset, recurrent infections requiring hospital-based management; and (ii) clinical characteristics indicative of syndromic or hereditary immunodeficiency and/or multisystem involvement. Exclusion criteria included isolated infectious illnesses without systemic manifestations; lack of consent for genetic evaluation; and suspected secondary immunodeficiency (e.g., malignancy or acquired etiologies). When available, parental samples were employed for segregation analysis.

Peripheral whole blood was procured from probands (and from parents when available). Genomic DNA was isolated using a standard salting-out protocol. Whole-genome sequencing (WGS) was performed on an Illumina NovaSeq 6000 platform (Illumina Inc., San Diego, CA, USA). Libraries were prepared using Illumina TruSeq DNA PCR-Free or TruSeq Nano DNA Library Prep Kits (Illumina Inc., San Diego, CA, USA) depending on DNA input, and sequenced according to the manufacturer’s instructions. Raw sequencing data were converted to FASTQ format using bcl2fastq software v2.20 (Illumina Inc., San Diego, CA, USA).

Bioinformatic processing included alignment to the human reference genome, variant calling, and annotation. Variants were filtered against population databases (e.g., gnomAD, 1000 Genomes) and annotated with Ensembl Variant Effect Predictor (Ensembl, Hinxton, Cambridge, UK). Interpretation adhered to ACMG/AMP guidelines; pathogenic, likely pathogenic, and selected variants of uncertain significance supported by multiple computational predictors were retained for clinical correlation. Candidate variants were validated by Sanger sequencing, and parental genotypes were evaluated for segregation when available. In silico pathogenicity assessment utilized standard algorithms, and molecular findings were correlated with clinical, immunologic, and available imaging and histopathology data.

### 3.2. Bioinformatics Analysis

Raw FASTQ files underwent quality control using FastQC v0.12.1 (Babraham Bioinformatics, Cambridge, UK). Paired-end sequences were aligned to the human reference genome (GRCh38 p14) using BWA-MEM (Broad Institute, Cambridge, MA, USA). Post-alignment processing followed GATK v4.4.0.0 Best Practices (Broad Institute, Cambridge, MA, USA): duplicate reads were marked with Picard MarkDuplicates (Broad Institute), base quality scores were recalibrated with GATK BaseRecalibrator, and variant calling was performed using GATK HaplotypeCaller in GVCF mode, followed by joint genotyping. Small-variant filtering was carried out using GATK VariantFiltration with parameters optimized for whole-genome sequencing data.

Functional annotation was performed using SnpEff (Computational Biology Group, Universidad Nacional de Quilmes, Buenos Aires, Argentina) and integrated with public variant databases, including dbSNP, 1000 Genomes, ESP6500, ClinVar, and dbNSFP. Gene names were standardized according to HGNC nomenclature. Population frequency filtering excluded variants with a minor allele frequency ≥0.01 in global or subpopulation datasets. Variants located in exonic or canonical splice regions and predicted to have protein-altering consequences (missense, nonsense, frameshift, or splice-site variants) were retained.

In silico pathogenicity prediction incorporated aggregated scores from dbNSFP v4.6a and additional predictive tools: SIFT v6.2.1 (J. Craig Venter Institute, Rockville, MD, USA), MutationTaster v2021 (Charité—Universitätsmedizin Berlin, Germany), MutationAssessor (Columbia University, New York, NY, USA), PrimateAI (Illumina Artificial Intelligence Lab, San Diego, CA, USA), DANN (University of Copenhagen, Denmark), and AlphaMissense (DeepMind Technologies, London, UK). Variants in genes relevant to the observed phenotypes (e.g., *FBLN5*, *TNFRSF13B*, and *ATM*) were prioritized and classified according to ACMG/AMP guidelines. Shortlisted variants were confirmed by Sanger sequencing, and segregation analysis was performed using parental DNA when available. Copy-number and structural variants were not systematically assessed and therefore could not be excluded. Molecular findings were correlated with clinical, immunologic, and available imaging and histopathology data.

### 3.3. Variant Interpretation/Information

All variants were described in accordance with Human Genome Variation Society (HGVS) recommendations (accessed 21 March 2024) using the following reference transcripts: *TNFRSF13B* NM_012452.3, *FBLN5* NM_006329.4, and *ATM* NM_000051.4. Variant classification followed the American College of Medical Genetics and Genomics/Association for Molecular Pathology (ACMG/AMP) five-tier system, incorporating evidence from population frequency, computational prediction, segregation data, prior literature, and functional annotation. Variants classified as pathogenic, likely pathogenic, or selected variants of uncertain significance were retained for clinical correlation and orthogonal validation.

Sanger validation and segregation. Candidate variants were corroborated through Sanger sequencing. Primers were conceived in VectorNTI (Thermo Fisher Scientific, Waltham, MA, USA) and the Oligomer application (PrimerDigital, Helsinki, Finland) to flank each locus by approximately 100–200 bp; primer sequences (5′→3′) and anticipated amplicon dimensions are enumerated in Table 1. Oligonucleotides were synthesized at the National Center for Biotechnology (Astana, Kazakhstan) utilizing an ASM-800 DNA synthesizer (Biosset, Novosibirsk, Russia). PCRs (25 µL) conventionally encompassed 10–50 ng genomic DNA, 1× buffer, 1.5–2.0 mM MgCl_2_, 0.2 mM dNTPs, 0.2 µM primers, and 1 U Taq polymerase; cycling parameters were 95 °C for 3 min; 35 cycles of 95 °C for 30 s, 58–62 °C for 30 s, 72 °C for 45–60 s; and 72 °C for 5 min. Amplicons were purified ExoSAP-IT (Applied Biosystems, Waltham, MA, USA) or equivalent and sequenced bidirectionally on an ABI 3500 capillary sequencer (Applied Biosystems, Waltham, MA, USA). Principal sequence analysis and contig assembly employed ContigExpress (Invitrogen, Waltham, MA, USA); chromatograms were scrutinized manually. Genotypes were designated only when bidirectional, high-quality (Phred ≥30) peaks were congruent with anticipated amplicon coordinates. When feasible, parental DNA was analyzed for segregation. For the FBLN5 c.53del locus, two alternative reverse primers were validated to ensure robust amplification (Table 1).

### 3.4. Ethical Compliance

All participants were thoroughly apprised of the aims, methodologies, and potential ramifications of the investigation, and their voluntary involvement was ensured through the acquisition of informed consent. For minors engaged in the research, informed consent was furnished by their guardians. The informed consent documentation and procedures were scrutinized and sanctioned by the Local Ethics Committee of the National Center for Biotechnology (Astana, Kazakhstan). The study conformed to the ethical tenets of the Declaration of Helsinki and the legal regulations of Kazakhstan, thus safeguarding academic freedom, intellectual integrity, and the entitlement to disseminate findings.

## 4. Results

### 4.1. Overview of the Study Cohort and Genetic Findings

Two unassociated male subjects were examined, each manifesting with early-onset, recurrent infections and multisystem involvement. The first was diagnosed with autosomal recessive cutis laxa type 1A (ARCL1A) attributable to a homozygous *FBLN5* variant, whereas the second was confirmed with ataxia–telangiectasia (A–T) resulting from a heterozygous *ATM* frameshift mutation. Both individuals underwent thorough clinical assessment and WGS, followed by Sanger validation, establishing the genetic basis of their disease. These cases highlight the clinical heterogeneity of IEIs and emphasize the role of genomic diagnostics in atypical presentations.

### 4.2. Case 1: Clinical, Immunological, Imaging, and Genetic Findings in FBLN5-Related Cutis Laxa Type 1A

**Figure 1:**
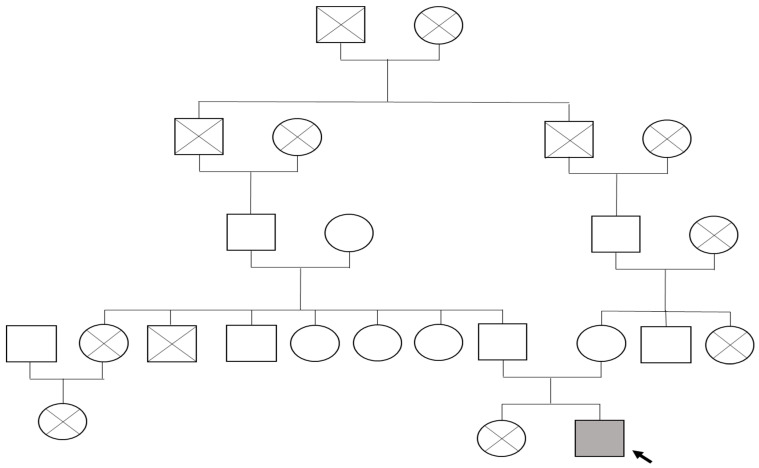
Family tree of proband with FBLN5-related cutis laxa type 1A: The proband (indicated by the black arrow) is represented by a shaded square (male) affected by autosomal recessive *FBLN5*-associated cutis laxa type 1A. Circles denote females, squares denote males, and diagonal lines indicate deceased individuals.

The pedigree illustrates the proband, the first surviving child of his mother’s second pregnancy (Figure 1). The first sibling (female) died in early infancy at three months due to pneumonia. Both parents are consanguineous, and the family history is notable for chronic urticaria in the father and bronchial asthma in the mother and maternal grandfather. No other congenital connective tissue or immunological disorders were reported in close relatives.

Since infancy, the patient exhibited recurrent colds with bronchopulmonary obstruction (BPO). The respiratory syndrome progressively deteriorated over time, with repeated pneumonias necessitating hospitalization. By 2018, chest CT imaging revealed pulmonary fibrosis. In recent years, the child developed monthly colds, recurrent severe BPO, and required hospitalization in May 2023 for acute respiratory decline. Despite regular inhaled therapy with Airtec 25/250 (Cipla Ltd., Mumbai, India) and Berodual (Boehringer Ingelheim International GmbH, Ingelheim am Rhein, Germany), his condition worsened, prompting the initiation of systemic corticosteroids (prednisolone 20 mg/day). He also received veroshpiron, sildenafil, and subcutaneous immunoglobulin (Gammanorm, Octapharma AB, Stockholm, Sweden) at 0.5 g/kg/month as lifelong therapy, in accordance with national treatment protocols.

The clinical diagnosis was consistent with autosomal recessive cutis laxa type 1A (FBLN5-related), complicated by pulmonary fibrosis, uncontrolled bronchial asthma, and immunodeficiency-related recurrent infections. Immunophenotyping (May 2022) revealed T-cell subset abnormalities, most notably elevated CD3+CD8+ frequencies and absolute counts with a relative reduction in CD3+CD4+ percentages (Table 2). Echocardiography in June 2022 showed right ventricular dilatation and elevated pulmonary artery systolic pressure (PASP 51–52 mmHg), with similar findings on follow-up in April 2023 (PASP 49 mmHg). Chest CT (March 2023) demonstrated fibrotic strands and pleural adhesions in the lower lobes bilaterally, along with parenchymal overaeration consistent with emphysematous and fibrotic remodeling.

Quantitative measurements of serum immunoglobulins (IgG, IgA, IgM) were not available in the medical records of this patient, as these investigations were not routinely performed in the regional hospital where he was initially managed. However, the diagnosis of immunodeficiency was supported by clinical features (recurrent sinopulmonary infections), immunophenotyping abnormalities, and a sustained clinical response to subcutaneous immunoglobulin replacement therapy.

Genetic testing by whole-genome sequencing (November 2024) identified two clinically relevant variants. In silico prediction tools supported the pathogenicity of the *FBLN5* c.53del (p.Pro18Glnfs*24*) variant: MutationTaster and SIFT both predicted a deleterious effect on protein function. The variant was absent in population genomic databases, including gnomAD, consistent with its rarity. Segregation analysis confirmed that both parents were heterozygous carriers, supporting autosomal recessive inheritance. Based on ACMG/AMP criteria and clinical presentation, this variant was classified as pathogenic. A heterozygous missense variant in *TNFRSF13B* (NM_012452.3:c.542C>A, p.Ala181Glu) was detected, along with a novel homozygous frameshift variant in *FBLN5* (c.53del, p.Pro18Glnfs24; genomic position chr14:91942925 TG>T). According to ACMG/AMP criteria, the *FBLN5* variant was classified as pathogenic, consistent with autosomal recessive cutis laxa type 1A, while the *TNFRSF13B* variant was considered a secondary finding of uncertain clinical significance. Sanger sequencing confirmed both variants and demonstrated parental segregation, thereby supporting the genetic diagnosis (Table 3).

Verification of the ascertained variants was executed through bidirectional Sanger sequencing, which substantiated the existence of both the *TNFRSF13B* and *FBLN5* modifications identified through whole-genome sequencing. Furthermore, segregation analysis employing DNA specimens from the proband’s progenitors illustrated heterozygous possession of the FBLN5 c.53del variant in both progenitors, in accordance with autosomal recessive inheritance. These findings affirmed the pathogenicity and familial transmission of the mutation (Table 4).

Grounded in the amalgamation of clinical manifestations, laboratory findings, and genetic evaluations, the patient received a diagnosis of lax skin syndrome (ICD-10: D80.8), further complicated by pulmonary fibrosis (J98.4), and persistent, moderate, uncontrolled bronchial asthma (J45.0). Management encompassed lifelong immunoglobulin replacement therapy utilizing subcutaneous Immunoglobulin G at a dosage of 0.5 g/kg/month. Administration was orchestrated 2–4 times per month, with dosage modifications contingent upon body mass and the incidence of exacerbations. In accordance with national directives regarding outpatient pharmacological provision (Order of the Ministry of Health of the Republic of Kazakhstan, 5 August 2021, No. ҚP ДCM-75), serum IgG and IgA concentrations were scrutinized prior to each administration to ascertain therapeutic efficacy and safety.

### 4.3. Case 2: Clinical, Dermatological, Neurological, Immunological, and Genetic Findings in Ataxia-Telangiectasia

Clinical assessment underscored the advancement of critical respiratory pathology, characterized by bilateral focal pneumonias and established bronchiectasis recorded on chest computed tomography in 2024, notwithstanding persistent immunoglobulin replacement therapy. Echocardiography verified supplementary systemic involvement, revealing mild valvular insufficiency concomitant with slightly elevated pulmonary artery pressures. These observations substantiated a diagnosis of advanced multisystem pathology with cardiopulmonary complications consequent to underlying immunodeficiency.

Chronic cutaneous involvement constituted a salient clinical feature, with persistent granulomatous lesions in the peri-auricular and submandibular regions documented from 2017 onwards. Histopathological evaluations across multiple biopsies produced disparate results, initially affirming a diagnosis of cutaneous tuberculosis due to acid-fast bacilli, subsequently suggesting sarcoidosis with non-caseating granulomas inclusive of Langhans giant cells, and ultimately collagenosis predicated on necrotizing granulomatous inflammation with fibrinoid necrosis. Notwithstanding early tuberculosis-directed therapy, subsequent biopsies were negative for *Mycobacterium tuberculosis*, favoring a process driven by immune dysregulation. Neurologically, cerebellar ataxia progressively exacerbated, and neuroimaging in 2020 unveiled structural abnormalities consistent with a variant of Dandy–Walker malformation, including enlargement of the cisterna magna, dilatation of the fourth ventricle, and diminished cerebellar hemispheric volume. Collectively, these findings accentuated the multisystem complexity of Ataxia–Telangiectasia, amalgamating immunological susceptibility, chronic dermatological pathology, and progressive cerebellar dysfunction.

Genetic testing by whole-genome sequencing in November 2024 identified a heterozygous frameshift variant in *ATM* (NM_000051.4:c.4828dup; genomic position chr11:108294976 C>CA), resulting in a premature stop codon (p.Arg1610Lysfs*3). This *ATM* variant has been previously reported in patients with classic ataxia–telangiectasia and is considered a recurrent pathogenic variant. It results in a premature termination codon consistent with loss of function, a known disease mechanism for *ATM* deficiency. The proband was heterozygous, but parental testing for segregation could not be completed due to the unavailability of parental samples. According to ACMG/AMP guidelines, this variant was classified as pathogenic based on strong evidence criteria. Sanger sequencing confirmed the finding and established its autosomal recessive inheritance pattern. These results provided definitive molecular confirmation of Ataxia–Telangiectasia in the proband (Table 5).

Confirmation of the identified *ATM* c.4828dup (p.Arg1610Lysfs*3) variant was achieved through bidirectional Sanger sequencing, which validated the heterozygous frameshift mutation in the proband. Parental genetic testing was proposed to assess segregation but was declined, precluding further analysis of inheritance in this family (Table 6).

Based on the amalgamation of clinical manifestation, laboratory analyses, and genetic verification, the patient was ascribed a diagnosis of Louis–Bar syndrome (Ataxia–telangiectasia, ICD-10: D80.4). Comorbid conditions encompassed bronchopneumonia, unspecified (J18.0), cutaneous sarcoidosis (D86.3), mucocutaneous candidiasis (B37.8), cerebellar ataxia with impaired DNA repair (G11.3), and chronic pancreatitis (K86.1). Management entailed lifelong immunoglobulin substitution therapy utilizing subcutaneous Immunoglobulin G at a dosage of 0.5 g/kg/week, administered weekly. Dosage modifications were executed in accordance with body mass and the existence of infectious exacerbations. This therapeutic regimen was instituted in alignment with the national directive on outpatient pharmaceutical provision (Order of the Ministry of Health of the Republic of Kazakhstan, 5 August 2021, No. ҚP ДCM-75).

## 5. Discussion

IEIs represent a heterogeneous group of hereditary disorders that often manifest with multisystem involvement and complex phenotypes. The two cases presented here, autosomal recessive cutis laxa type 1A caused by a novel *FBLN5* variant and ataxia telangiectasia associated with a pathogenic *ATM* variant, illustrate the diagnostic difficulties related to overlapping clinical features. Respiratory, connective tissue, dermatological, and neurological manifestations may obscure the underlying immunogenetic cause and delay accurate diagnosis [14]. In both instances, whole genome sequencing combined with Sanger confirmation provided definitive molecular diagnosis and enabled appropriate management. Reporting such genetically verified IEIs from Central Asia, a region with limited genomic data, contributes to closing regional knowledge gaps and expands the clinical and genetic spectrum of these disorders [1].

Autosomal recessive cutis laxa type 1 is primarily associated with abnormalities in connective tissue and elastic fiber formation, resulting in arterial tortuosity, aneurysms, and pulmonary or cardiovascular complications [15]. Mutations in *FBLN5* disrupt elastic fiber assembly by altering interactions with integrins, fibrillin, and lysyl oxidases, compromising tissue elasticity and structural integrity [16]. Previously reported *FBLN5*-related cases were characterized by lax skin, cardiovascular anomalies, and hernias, while pulmonary fibrosis is rare. Our patient presented with pulmonary fibrosis and recurrent infections, broadening the recognized clinical spectrum of *FBLN5*-related cutis laxa [15]. The identified homozygous *FBLN5* c.53del frameshift variant was absent from population databases, representing a novel pathogenic allele. The *FBLN5* c.53del variant predicts a frameshift with an early premature stop codon that is consistent with loss of function in a gene where haploinsufficiency and null alleles are an established disease mechanism [15,16,17]. In silico prediction supported a deleterious effect of the frameshift with conservation at the affected region and high pathogenicity scores, which is consistent with *FBLN5* loss of function as the established disease mechanism [15,16,17]. The allele was absent from gnomAD and internal datasets, which supports rarity. The homozygous state occurred in a consanguineous family, and the proband’s phenotype aligns with connective tissue and pulmonary involvement classically attributed to *FBLN5* deficiency [15,16,17]. Although segregation analysis beyond the proband was not feasible, the combination of a truncating effect, absence from population databases, and phenotype-to-gene concordance supports likely pathogenicity. Consanguinity within the family underscores an important epidemiological factor contributing to the burden of recessive diseases in this region and highlights the importance of genetic counseling [16,17].

Ataxia telangiectasia is characterized by progressive neurodegeneration, immunodeficiency, and increased malignancy risk. The *ATM* c.4828dup frameshift variant identified in our patient was not reported in gnomAD or ClinVar and resulted in a truncated protein consistent with loss of function. The variant is therefore considered novel in the context of pediatric ataxia telangiectasia, and the heterozygous genotype together with the clinical phenotype supports autosomal recessive inheritance despite the lack of parental segregation due to declined testing. To our knowledge, this *ATM* frameshift has not been previously described in the pediatric A-T literature as of the time of writing. The patient exhibited granulomatous dermatitis and chronic pancreatitis, features rarely described in children with *ATM* deficiency, emphasizing the disorder’s phenotypic diversity [11,18,19].

Our findings are consistent with previous pediatric reports describing wide clinical variability in *FBLN5* and *ATM* related disorders [20,21,22,23,24], yet they extend the spectrum in two clinically relevant ways. In *FBLN5*-related disease, most pediatric descriptions emphasize cutaneous laxity with cardiovascular and hernia phenotypes, whereas progressive pulmonary fibrosis in childhood appears uncommon; the present case contributes additional evidence that lung parenchymal involvement can be an early and clinically significant feature [20,21,22,23,24]. In *ATM* deficiency, granulomatous dermatitis and chronic pancreatitis have been only sporadically reported in children; their co-occurrence in our patient underscores the breadth of inflammatory manifestations and may inform anticipatory guidance [20,21,22,23,24]. Management in resource-limited settings focuses on immunoglobulin replacement, infection prophylaxis, physiotherapy, and avoidance of ionizing radiation, with close monitoring of pulmonary and cardiovascular systems. In resource-limited settings, core management includes long-term immunoglobulin replacement, vaccination review with avoidance of live vaccines where appropriate, aggressive pulmonary hygiene and physiotherapy, early treatment of bacterial exacerbations, and strict avoidance of ionizing radiation in *ATM* deficiency, with multidisciplinary surveillance of pulmonary and cardiovascular complications [1,2,25]. Where feasible, enrollment in ESID or allied registries facilitates standardized follow-up, benchmarking of outcomes, and access to expert networks [25].

Beyond the main causal variants, our genomic analysis additionally identified a heterozygous variant in *TNFRSF13B*, which encodes the transmembrane activator and calcium-modulator and cyclophilin-ligand interactor (TACI), a BAFF/APRIL receptor essential for B-cell maturation, T-independent responses, and class switch recombination. Pathogenic and likely pathogenic *TNFRSF13B* alleles, most classically C104R and A181E, have been associated with common variable immunodeficiency and IgA deficiency, although penetrance is incomplete and monoallelic carriage is frequent among healthy individuals, indicating that many heterozygous changes act as disease-modifying risk alleles rather than fully penetrant monogenic causes [26,27,28]. Clinically, *TNFRSF13B* carriers demonstrate increased frequencies of autoimmunity, granulomatous inflammation, and lymphoproliferation even when hypogammaglobulinemia is mild or delayed [26,27,28]. In our context, the detected *TNFRSF13B* variant is most plausibly a modifier that may augment susceptibility to immune dysregulation without supplanting the primary diagnoses driven by *FBLN5* and *ATM*. We therefore recommend longitudinal monitoring of immunoglobulin levels (IgG/IgA), specific antibody responses, and autoimmune or granulomatous features, with reclassification of the variant as emerging functional and population data warrant [29].

Both patients exhibited early-onset recurrent infections and progressive multisystem involvement affecting pulmonary, cardiovascular, dermatological, and neurological systems. These parallels highlight the clinical heterogeneity of IEIs, where distinct genetic mutations can produce overlapping phenotypes. Long-term immunoglobulin replacement therapy proved essential to control infection burden and preserve organ function. Genetic confirmation by whole genome sequencing and Sanger sequencing reduced diagnostic uncertainty and facilitated tailored management. The clinical complexity of both patients emphasizes the necessity of coordinated care involving pulmonologists, neurologists, dermatologists, cardiologists, and clinical geneticists to optimize outcomes.

The two cases also illustrate the regional context of IEIs in Kazakhstan and neighboring Central Asian countries, where these disorders remain underdiagnosed due to limited access to molecular testing, delayed specialist referral, and the confounding nature of overlapping syndromic presentations. Elevated rates of consanguinity in certain populations further increase the prevalence of autosomal recessive diseases. Enhancing clinician awareness, expanding national diagnostic capacities, and establishing comprehensive IEI registries are crucial steps toward earlier diagnosis and improved care. Documenting rare genetically confirmed cases not only aids differential diagnosis but also strengthens the foundation for regional collaboration and public health policy development [1,2].

Although this study describes only two cases, it provides valuable insights into the molecular and clinical diversity of rare IEIs. The discovery of a novel *FBLN5* variant expands the mutational landscape of cutis laxa type 1A, while the *ATM* variant broadens phenotypic recognition of ataxia telangiectasia. Integrating genetic, clinical, and imaging data allowed detailed genotype-to-phenotype correlation. The absence of functional assays and segregation confirmation in one family remains a study limitation. Future research should focus on functional validation, expanded genetic screening, and the inclusion of Central Asian cohorts in international registries to improve epidemiological understanding and patient outcomes.

## 6. Conclusions

The two cases presented in this study illustrate the complexity and clinical diversity of inborn errors of immunity and emphasize the crucial role of genomic diagnostics in achieving accurate diagnoses. The identification of a novel homozygous *FBLN5*:c.53del frameshift variant in cutis laxa type 1A and a heterozygous *ATM*:c.4828dup frameshift variant in ataxia telangiectasia demonstrates the value of whole genome sequencing followed by Sanger confirmation in clarifying atypical and overlapping phenotypes. Both patients experienced early-onset infections, progressive multisystem involvement, and a lifelong need for immunoglobulin replacement therapy, reflecting common challenges in the management of rare immunodeficiencies.

These findings also highlight the occurrence of uncommon features such as pulmonary fibrosis, severe asthma, granulomatous skin lesions, and chronic pancreatitis, which broaden the known clinical spectrum of these disorders. From a regional perspective, the cases underline the limited recognition of rare immunogenetic diseases in Kazakhstan and Central Asia due to restricted genomic resources, delayed specialist referral, and high rates of consanguinity.

In summary, comprehensive clinical assessment combined with advanced genomic diagnostics remains essential for reducing diagnostic delay, optimizing therapeutic strategies, and guiding genetic counseling. By contributing novel genetic and clinical data, this study strengthens global understanding of phenotypic variability in rare immunodeficiencies and provides a foundation for improving patient care in resource-limited settings.

## Figures and Tables

**Table 1:** Designed primers used for the validation of the identified variants.

Gene	Mutation	Primer Direction	Primer Sequence (5′ to 3′)
*ATM*	c.4828dup	Forward	AAGTTTTATTTCACAGGCTTAACC
		Reverse	GACCAGTTAGGACCAGTTTTAC
*TNFRSF13B*	c.542C>A	Forward	CTAATGACGGGAAGAGAAGAAGG
		Reverse	CTAGAAGCAGGGGCAGTGA
*FBLN5*	c.53del	Forward	AGGAGGCAAGTAGAAGAGGT
		Reverse	GACTGGGAGTCAGGAAAC
		Reverse	GGAATCACGCACACAGGTA

**Table 2:** Immunophenotyping results of proband (May 2022).

Parameter	Result	Reference Range
CD3+CD19− (%)	73.10	66.00–76.00
CD3+CD19− (abs, ×10^9^/L)	2.36	1.40–2.00
CD3+CD4+ (%)	32.60	33.00–41.00
CD3+CD4+ (abs, ×10^9^/L)	1.05	0.70–1.10
CD3+CD8+ (%)	38.60	27.00–35.00
CD3+CD8+ (abs, ×10^9^/L)	1.24	0.60–0.90

**Table 3:** Results of whole-genome sequencing of the proband.

Variant Detected	Gene	Zygosity	Amino Acid Change	Inheritance	Classification
NM_012452.3:c.542C>A	*TNFRSF13B*	Heterozygous	p.Ala181Glu	AR/AD	Pathogenic (PM2, PM5, PP4, PP5)
NM_006329.4:c.53del; chr14:91942925 TG>T (novel)	*FBLN5*	Homozygous	p.Pro18Glnfs*24	AR	Pathogenic (PVS1, PM2, PP4)

“*” indicates a premature stop codon.

**Table 4:** Results of Sanger sequencing of the proband and parents.

Gene/Variant	Sample	Genotype	Sequencing Data
*TNFRSF13B* c.542C>A (p.Ala181Glu)	Proband	G/T	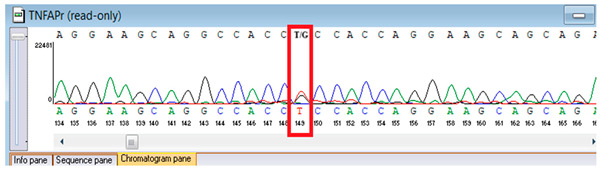
	Father	G/G	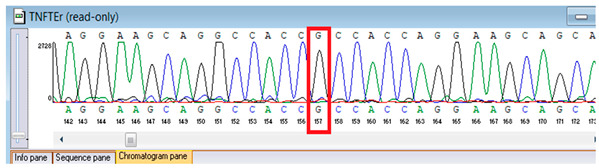
	Mother	G/T	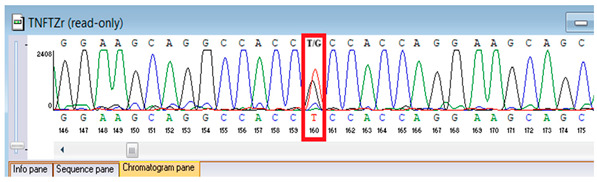
*FBLN5* NM_006329.4:c.53del (chr14:91942925 TG>T; p.Pro18Glnfs*24)	Proband	T/T	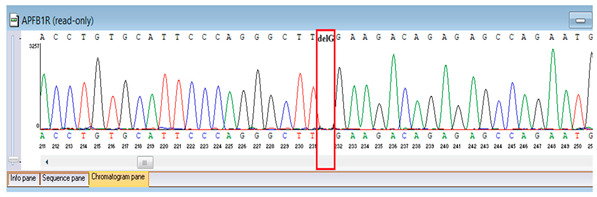
	Father	TG/T	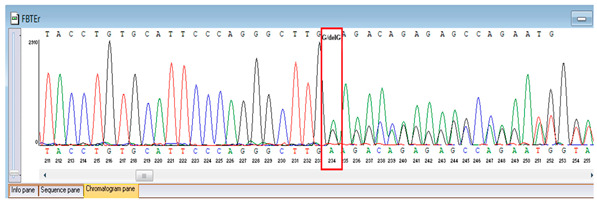
	Mother	TG/T	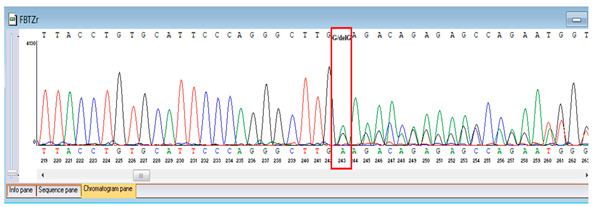

Representative chromatograms illustrate genotypes for *TNFRSF13B* c.542C>A (p.Ala181Glu) and *FBLN5* c.53del (p.Pro18Glnfs*24) variants. The red boxes highlight the variant nucleotide positions detected in the proband and parents. Bidirectional sequencing confirmed the heterozygous or homozygous states as indicated. Sequence data are aligned to *TNFRSF13B* (NM_012452.3) and *FBLN5* (NM_006329.4) reference transcripts. “*” indicates a premature stop codon.

**Table 5:** Results of whole-genome sequencing of the patient.

Variant (HGVS/Genomic)	Gene	Zygosity	Amino Acid Change	Inheritance	Classification
NM_000051.4:c.4828dup; chr11:108294976 C>CA	*ATM*	Heterozygous	p.Arg1610Lysfs*3 (frameshift)	AR	Pathogenic (PVS1, PM2, PP4)

“*” indicates a premature stop codon.

**Table 6:** Results of Sanger sequencing of the proband.

Gene/Variant	Sample	Genotype	Sequencing Data
ATM:c.4828dup chr11-108294976 C>CA p.Arg1610Lysfs*3	Proband	CA/CA	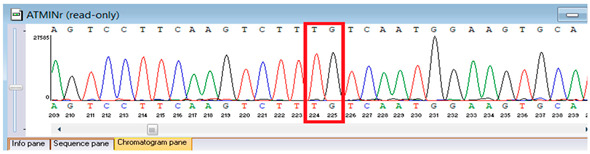

“*” indicates a premature stop codon.

## Data Availability

The authors confirm that the data supporting the findings of this study are available within the article.

## Abbreviations

The following abbreviations are used in this manuscript:

ACMG	American College of Medical Genetics and Genomics
ADCL	Autosomal dominant cutis laxa
AMP	Association for Molecular Pathology
AR	Autosomal recessive
BCL	Base call file
CONSORT	Consolidated Standards of Reporting Trials
CT	Computed tomography
DNA	Deoxyribonucleic acid
dNTPs	Deoxynucleotide triphosphates
ELN	Elastin gene
Gdna	Genomic DNA
HGNC	HUGO Gene Nomenclature Committee
HGVS	Human Genome Variation Society
IEI	Inborn error of immunity
IUIS	International Union of Immunological Societies
MRI	Magnetic resonance imaging
NGS	Next-generation sequencing
PASP	Pulmonary artery systolic pressure
PCR	Polymerase chain reaction
PICU	Pediatric intensive care unit
SNP	Single-nucleotide polymorphism
SVAS	Supravalvular aortic stenosis
URDS	Urban–Rifkin–Davis syndrome
WES	Whole-exome sequencing
WGS	Whole-genome sequencing
